# Linguistic and clinical validation of the acute cystitis symptom score in German-speaking Swiss women with acute cystitis

**DOI:** 10.1007/s00192-021-04864-1

**Published:** 2021-06-25

**Authors:** Jakhongir F. Alidjanov, Ulugbek A. Khudaybergenov, Bekhzod A. Ayubov, Adrian Pilatz, Stefan Mohr, Julia C. Münst, Olivia N. Ziviello Yuen, Sabine Pilatz, Corina Christmann, Florian Dittmar, Nodir M. Mirsaidov, Mareike Buch-Heberling, Kurt G. Naber, Truls E. Bjerklund Johansen, Florian M. E. Wagenlehner

**Affiliations:** 1 Department of Urology, Pediatric Urology and Andrology, Justus-Liebig University of Giessen, Rudolph-Buchheim str. 7, 35392 Giessen, Hessen Germany; 2 Department of Urology, Tashkent Medical Academy, Tashkent, Uzbekistan; 3 Department of Urological Surgery, Republican Specialized Scientific-Practical Medical Center of Urology, Tashkent, Uzbekistan; 4 Department of Urology, Pediatric Urology and Andrology, Justus Liebig University of Giessen, Giessen, Hessen Germany; 5 Department of Obstetrics and Gynecology, Inselspital, Bern University Hospital, University of Bern, Bern, Switzerland; 6 Department of Gynaecology, Kantonsspital Frauenfeld, Frauenfeld, Switzerland; 7 Blasenzentrum Zürich, Zurich, Switzerland; 8 Practice for general medicine, Lollar, Germany; 9 Department of Obstetrics and Gynecology, Cantonal Hospital of Lucerne, Lucerne, Switzerland; 10 Department of Urology, Technical University of Munich, Munich, Germany; 11 Department of Urology of Oslo University Hospital, University of Oslo, Oslo, Norway; 12 Institute of Clinical Medicine, University of Aarhus, Aarhus, Denmark

**Keywords:** Acute cystitis, Urinary tract infection, Questionnaire, Diagnosis, Female, Patient-reported outcome

## Abstract

**Introduction and hypothesis:**

The Global Prevalence Study of Infections in Urinary tract in Community Setting (GPIU.COM) includes epidemiological aspects of acute cystitis (AC) in women in Germany and Switzerland. The primary study relates to the German version of the Acute Cystitis Symptom Score (ACSS), a self-reporting questionnaire for self-diagnosis and monitoring the symptomatic course of AC in women. The current study aimed to analyze the validity and reliability of the German ACSS in German-speaking female patients with AC in Switzerland.

**Methods:**

Anonymized patient data were collected and analyzed from women with AC at the first visit (diagnosis) and follow-up visits as baseline and controls, respectively. Data from 97 patients with a median age of 41 years underwent analysis. Psychometric and diagnostic characteristics of the ACSS were measured and statistically analyzed.

**Results:**

Average internal consistency of the ACSS resulted in a Cronbach’s alpha (95% CI) of 0.86 (0.83; 0.89) and did not differ significantly between the Swiss and German cohorts. Diagnostic values of the ACSS for the Swiss cohort were relatively lower than for the German cohort, possible due to discrepancies between definitions of UTI in national guidelines.

**Conclusions:**

The analysis showed that the German version of the ACSS is also suitable for use in the German-speaking female population of Switzerland. Minor differences in definitions of AC between German and Swiss guidelines explain the observed discrepancies in diagnostic values of the ACSS between cohorts.

**Supplementary Information:**

The online version contains supplementary material available at 10.1007/s00192-021-04864-1.

## Introduction

Uncomplicated urinary tract infections (uUTIs) are common in the global female population, accounting for at least one symptomatic episode of acute cystitis during a lifetime, and about one-third of women first experience an acute episode before the age of 24 [1, 2].

Recent publications on complicated (cUTIs) and hospital-acquired urinary tract infections (HAUTIs) report a drastic increase in antimicrobial resistance of uropathogens [3–5]. At the same time, the inconsistency of national and international guidelines concerning definitions and diagnostic criteria for UTI and the fact that uUTIs have long been overlooked have probably led to current obstacles to performing adequate research on the global prevalence of uUTI [6–11].

The Global Prevalence Study of Infections in Urinary tract in a Community setting (GPIU.COM) is an epidemiological survey to explore the prevalence of AC in a healthcare community setting. This study was initiated by the European Section for Infections in Urology (ESIU), which is affiliated with the European Association of Urology (EAU), and aimed to highlight contemporary aspects of uUTI in women, such as the prevalence of AC and its relevant risk factors, the most common causative uropathogens and their resistance to commonly prescribed antimicrobial agents, etc. This part of the GPIU.COM-Study aimed to test the strategy of unified standards and tools for possible “pros and cons” in a German-speaking population and included clinics in Germany and Switzerland. The German version of the Acute Cystitis Symptom Score (ACSS) was used as the primary tool for recruitment for the symptomatic diagnosis of AC in female patients [12].

The current part of the GPIU.COM-Study aimed to test the hypothesis of the equality of the psychometric parameters and diagnostic values of the German language version of the ACSS in German-speaking female patients in Switzerland and Germany.

## Materials and methods

### Study design

The GPIU.COM-Study was initiated and designed by the Clinic of Urology, Pediatric Urology and Andrology of the Justus-Liebig University Giessen, Germany, as a prospective, observational, multinational internet-based audit trial on the epidemiology of AC in women. The primary study protocol was approved by the Ethics Committee of the Justus-Liebig University of Giessen, Germany (ethical approval code: AZ.:10/15, August 4, 2015). This part of the GPIU.COM-Study was restricted to selected clinics in Germany and Switzerland under the scientific direction of the ESIU.

### Study tool

The ACSS is a self-reporting symptom questionnaire for women suffering from AC for the assessment of the presence and severity of symptoms related to AC and monitoring of the course of symptomatic episodes of AC. The development, structure and validation of the ACSS were described in detail previously [13].

The process of translation and validation of the ACSS from the source Uzbek language into the German target language was performed according to international guidelines and recommendations including steps, such as forward and backward translations, revision, correction, cognitive assessment, pilot clinical validation, additional reconciliation and further corrections [12, 14–17].

### Study subjects and procedures

Female patients at the age of 16 and older admitted to the clinics with at least two clinical symptoms suspicious for AC (e.g., dysuria, frequency, urgency) were recruited to participate in the study. Written informed consent was obtained from all respondents before the start of the study procedures. At the first admission to the physician’s office (baseline visit) patients were requested to fill in the registry form with questions on demographic characteristics, history of previous symptomatic episodes of lower urinary tract infections (LUTI), medications and hospitalizations in the preceding 12 months, if any. The presence and severity of symptoms of AC and relevant signs and symptoms were assessed by patients using the first, diagnostic (part-A) form of the German ACSS [15]. The additional information about the overall health-related quality of life was collected, using the validated German three-level version of the EuroQoL-5 Dimension (EQ-5D-3L) Health Questionnaire [18, 19]. The risk factors of patients to develop more severe outcomes were classified by the physician according to the ORENUC system [20]. The lower urinary tract infection recurrence risk nomogram (LUTIRE) by Cai et al. was used to assess the risk of recurrence of symptomatic UTI and to support additional demographic data from patients [21]. Urine samples were taken from patients for dipstick analysis, and urine culture was performed when appropriate.

At the end of the visit, the attending physician prescribed appropriate medical treatment and preventive measures to the patients according to the recommendations of local and international guidelines. Patients were invited for a follow-up visit 5–10 days after the baseline visit. In case a follow-up visit was not possible, patients were requested to complete the follow-up (part-B) form of the ACSS and EQ-5D-3L Health Questionnaire and return them by mail to the attending physician.

If present at the follow-up visits, patients were requested to pass a mid-stream urine for dipstick test and urine culture, when available. The change in patient’s clinical condition during therapy was independently assessed by the attending physician at the follow-up visit, using a predefined “clinical scenario” scale graded as: “complete resolution of lower UTI,” “improvement in symptoms,” “no change in condition” and “deterioration of symptoms.” Obtained data were entered into a database using prespecified case report forms. Only the cases with non-missing demographic and questionnaire data were included in the study.

### External control population as a reference for the testing the study hypothesis

As arbitrate control group, we created a virtual “International” cohort consisted of patients who completed the ACSS in their native language. Patients for this cohort were randomly selected from our database comprising data from previous studies in different countries.

### Data processing

Since the GPIU.COM Study included only symptomatic patients, the data obtained at the baseline visit were used to define the “positive” outcome, and the data obtained at the follow-up visit(s) were used to define “negative” (control) outcome. From these data, the psychometric reliability and diagnostic value of the German ACSS for Switzerland were assessed.

Graded interval variables of the clinical scenario were converted to ordinal data as follows: 0 = complete resolution of lower UTI; 1 = improvement in symptoms; 2 = for no change in condition; 3 = deterioration of symptoms.

Dichotomous variables were labeled as 0 for “negative/not match” and 1 for “positive/match.” Missing numerical or ordinal variables underwent multiple imputations by the median. Missing categorical variables were not imputed to avoid biases.

### Statistical analysis

To test the hypothesis about the equality of performance of the German ACSS among the German-speaking women both in Germany and Switzerland, the reliability, validity and diagnostic values of the German version of ACSS were analyzed and compared between German and Swiss cohorts, using the “International” cohort as an arbitrary comparator.

Normality of distributions and linearity and homoscedasticity of data were tested visually (using histograms, normal Q-Q plots, etc.) and mathematically (using Shapiro-Wilk and Levene’s tests) [22, 23]. Continuous variables were presented in averages with 95% confidence intervals (CI), medians and interquartile ranges (IQR).

Reliability of the ACSS and its domains was assessed via internal consistency of the items and represented using Cronbach’s alpha and split-half reliability [24].

Convergent validity was assessed via the measurement of the strength of the associations between ACSS item scores and respective items of the validated German version of the EQ-5D-3L [18, 19].

Responsiveness of the ACSS domains was measured by the strength of the relationships between the difference in summary scores at baseline and follow-up visits and the scores of “clinical scenario,” assessed by attending physicians at the follow-up visits.

Responsiveness of the “Dynamics” domain was separately assessed by the calculation of the strength of the relationships between its levels and the physician’s independent assessment of the change in the patient’s condition using the scores of “clinical scenario.”

Diagnostic values of the domains and items of the ACSS were assessed by measurement of sensitivity, specificity, positive and negative likelihood ratios, diagnostic odds ratio (DOR), Youden’s index and ROC curve analyses.

Discriminative ability was assessed by comparing the scores of the respective items and summary scores of the domains at the baseline visit with those at the follow-up visits after therapy.

A comparative analysis of the independent continuous variables was performed using a two-sided Student *t*-test with the Welch correction in cases of inequality of variances when comparing two cohorts (e.g., Swiss vs. German) and Kruskal-Wallis test when comparing three cohorts (Swiss, German and International) [25, 26].

Categorical variables were presented in proportions and compared with McNemar’s test [27]. Ordinal and interval variables were compared using the Wilcoxon signed-rank test [28]. The strength of associations was assessed using Pearson’s correlation coefficient (*r*) for numerical variables and nonparametric Spearman’s rank correlation (*rho*) for interval variables [29, 30]. Statistical significance was set at 0.05.

R v.3.5.2 with in-built and additional packages was used for the analysis and graphical representation of the results [31–34].

## Results

### The German ACSS as a study tool

The German version of the ACSS is presented as a Suppl. Fig. 1 [12, 14].

### Study population

Among the data from 109 patients inputted to the online database, 97 (89.0%) cases from five medical institutions^1^ were considered valid and included in further analysis. The median age (IQR) of the included patients was 41.0 (28.0–57.0) ranging from 17 to 83 years.

Of the selected 97 patients, 71 (73.2%) were from Switzerland and 26 (26.8%) from Germany. Further available demographic data and their differences between cohorts are presented in Table 1. Except for the presence of pyuria, summary scores of the ACSS domains and the proportions of cases with available follow-up data, both cohorts were homogeneous (Table 1).

**Table 1:** Demographic and clinical characteristics of the patients included in the analysis

Parameter	Total cohort (%)	Swiss cohort	German cohort	*P* value*
Number, *n* (%)	97 (100)	71 (73.2)	26 (26.8)	n.a.
Age, years, median (IQR)	41 (28–57)	43 (27.5–59.5)	39 (28.0–49.5)	0.323
Body-mass index, median (IQR)	24.1 (21.2–27.7)	24.2 (21.2–27.6)	23.8 (20.6–28.2)	0.849
History of antibiotics in 3 months, *n* (%)	30 (30.9)	24 (33.8)	6 (23.1)	0.445
Number of symptomatic episodes per year, median (IQR)	2.0 (1.0–2.0)	2.0 (2.0–2.0)	2.0 (0.3–2.0)	0.064
Cases with or without risk factors for UTI according to the ORENUC system				
Cases with no known risk factors for UTI (O), *n* (%)	70 (72.2)	54 (76.1)	16 (61.5)	0.247
Risk of recurrent UTIs but without risk of a more severe outcome (R), *n* (%)	22 (22.7)	15 (21.1)	7 (26.9)	0.741
Extraurogenital risk factors (E), *n* (%)	2 (2.1)	1 (1.4)	1 (3.8)	n.a.
Relevant nephropathic diseases (*N*), *n* (%)	2 (2.1)	0 (0.0)	2 (7.7)	0.120
Urologic resolvable risk factors (U), *n* (%)	4 (4.1)	3 (4.2)	1 (3.8)	1.000
Permanent external urinary catheter and unresolved urologic risk factors (C), *n* (%)	0 (0.0)	0 (0.0)	0 (0.0)	n.a.
Cases according to factors for recurrent UTI following Cai’s nomogram				
Number of sexual partners within the last year				
One sexual partner, *n* (%)	81 (83.5)	61 (85.9)	20 (76.9)	0.776
Two sexual partners, *n* (%)	7 (7.2)	5 (7.0)	2 (7.7)	1.000
More than two sexual partners, *n* (%)	5 (5.2)	3 (4.2)	2 (7.7)	0.826
Bowel function				
Normal bowel function, *n* (%)	74 (76.3)	53 (74.6)	21 (80.8)	0.410
Predisposed to constipation, *n* (%)	14 (14.4)	12 (16.9)	2 (7.7)	0.461
Predisposed to diarrhea, *n* (%)	5 (5.2)	4 (5.6)	1 (3.8)	1.000
Type of pathogens, isolated at the last episode of UTI				
Gram (−) pathogens, isolated at the last episode of UTI, *n* (%)	19 (19.6)	14 (19.7)	5 (19.2)	1.000
Gram (+) pathogens, isolated at the last episode of UTI, *n* (%)	4 (4.1)	3 (4.2)	1 (3.8)	1.000
Pathogens, isolated at the last episode of UTI are unknown, *n* (%)	70 (72.2)	52 (73.2)	18 (69.2)	1.000
Hormonal status at the time of visit				
Premenopausal, *n* (%)	61 (62.9)	44 (62.0)	17 (65.4)	0.705
Postmenopausal, *n* (%)	32 (33.0)	25 (35.2)	7 (26.9)	0.705
Number of symptomatic episodes of UTI within the last year				
Up to 2 episodes, *n* (%)	51 (52.6)	34 (47.9)	17 (65.4)	0.112
More than 2 episodes, *n* (%)	42 (43.3)	35 (49.3)	7 (26.9)	0.112
Preceding antimicrobial therapy, *n* (%)	25 (25.8)	14 (19.7)	11 (42.3)	0.030
Probability of recurrence of UTI within next 12 months, median (IQR)	0.3 (0.2–0.5)	0.3 (0.2–0.5)	0.3 (0.3–0.4)	0.882
Summary scores of the domains of the ACSS				
Summary “Typical” score at baseline visit, median (IQR)	8.0 (5.0–12.0)	8.0 (5.0–9.8)	11 (7.8–13.2)	0.015
Summary “Differential” score at baseline visit, median (IQR)	0.0 (0.0–2.0)	0.0 (0.0–1.0)	2.0 (1.0–3.0)	< 0.001
Summary “QoL” score at baseline visit, median (IQR)	5.0 (3.0–6.0)	4.0 (2.3–6.0)	6.0 (4.0–7.0)	0.008
Summary score of the entire ACSS	14.0 (10.0–18.0)	12.0 (9.0–16.0)	17 (13.3–22.0)	0.001
Cases, attempted to treat previously, *n* (%)	28 (28.9)	24 (33.8)	4 (15.4)	0.128
Urine dipstick positive for WBC, *n* (%)	81 (83.5)	56 (78.9)	25 (96.2)	0.027
Pyuria; moderate-to-large amount of WBC urine dipstick, *n* (%)	57 (58.8)	34 (47.9)	23 (88.5)	0.036
Urine dipstick test positive for nitrite, *n* (%)	32 (33.0)	23 (32.4)	9 (34.6)	0.932
Positive urine culture, *n* (%)	63 (64.9)	48 (67.6)	15 (57.7)	1.000
Patients, having menstruations at baseline, *n* (%)	10 (10.3)	8 (11.3)	2 (7.7)	0.876
Patients, presented with premenstrual symptoms, *n* (%)	6 (6.2)	2 (2.8)	4 (15.4)	0.063
Patients, presented with symptoms of menopause, *n* (%)	10 (10.3)	7 (9.9)	3 (11.5)	1.000
Patients with diabetes mellitus, *n* (%)	5 (5.2)	3 (4.2)	2 (7.7)	0.814
Patients followed up to the more than one visit, *n* (%)	41 (42.3)	24 (33.8)	17 (65.4)	0.011

*Swiss cohort vs. German cohort

Of the 28 patients treated by antimicrobials before the baseline visit, 21 (21.6%) received mono-antimicrobial therapy, 7 (7.2%) had received the combined antimicrobial therapy (more than 1 antimicrobial agent), and for 69 (71.1%) the type of therapy was unknown. The most used antimicrobial agent before the baseline visit was fosfomycin (18.6%).

Urine dipstick test resulted in positive leukocyturia (≥ 25 leucocytes per ml) in 81 (83.5%), nitrite test was positive for 32 (33.0%), and urine culture was positive (≥ 1000 CFU/ml) for 63 (64.9%) of patients (Table 1).

Follow-up data were available for 41 (42.3%) of 97 patients: 24 in the Swiss cohort (33.8%) and 17 in the German cohort (65.4%). Of these, a complete resolution of LUTI was noted in 16 (43.9%), improvement in symptoms in 15 (36.6%), deterioration of symptoms in 1 (2.4%) and no change in symptoms was in 9 (22.0%).

The reference “International” cohort consisted of 71 patients, randomly selected from our database. The flowchart of the preparation and the selection process is presented in Suppl. Fig. 2.

### Psychometric reliability

Test for the internal consistency of the German ACSS identified the following Cronbach’s alpha coefficients [95% CI] in the study cohort: 0.82 [0.78; 0.87] for the “Typical” domain, 0.32 [0.15; 0.50] for the “Differential” domain, 0.91 [0.89; 0.94] for the “QoL” domain, and in 0.86 [0.83; 0.89] for the entire ACSS. These values were lower for the Swiss cohort, compared to the German cohort, though the difference was not statistically significant (Suppl. Table 1A).

**Fig. 1:**
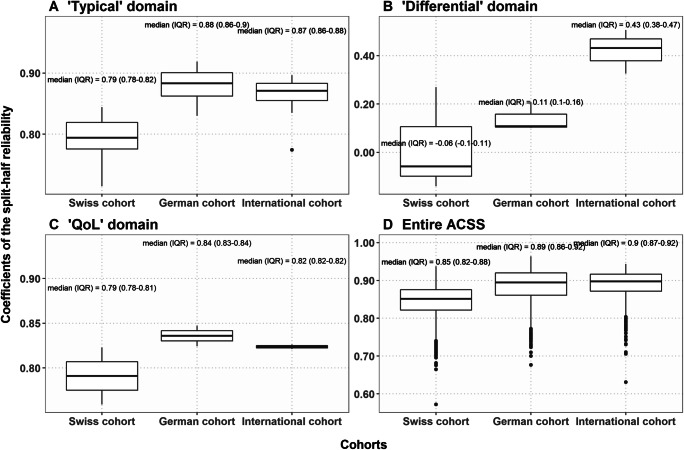
**A**–**D** Coefficients of split-half reliability for summary scores of the ACSS domains for Swiss and German cohorts of the study and the reference “International” cohort

Cronbach’s alpha coefficients of the ACSS domains in the “International” cohort were close to those in the German cohort of the study and did not differ significantly from the Cronbach’s alpha coefficients of the Swiss cohort (Suppl. Table 1 B). The values of split-half reliability for the “Typical” domain and entire ACSS were significantly lower in the Swiss cohort compared to the German and International cohorts, which in turn did not differ significantly between each other (Fig. 1).

### Convergent validity

The graphical illustration of the associations between the domains of the ACSS and items of the EQ-5D-3L Health Questionnaire (Suppl. Fig. 3A-D) demonstrates that patients who scored higher on the items of the “Typical” domain of the ACSS were more predisposed to have problems with self-care and usual activities (Suppl. Fig. 3 A), whereas patients who gained higher scores in the “QoL” domain of the ACSS were additionally predisposed to feel a greater sense of pain or discomfort, have problems with mobility and be more susceptible to anxiety and depression (Suppl. Fig. 3 C).

All items of the ACSS for both cohorts had a significant negative correlation with the General Health Status of the EQ-5D-3L. As expected, the “Differential” domain of the ACSS had no significant correlation with the items of the EQ-5D-3L questionnaire, except for the item of “General Health Status” (Suppl. Table 2).

### Responsiveness

**Fig. 2:**
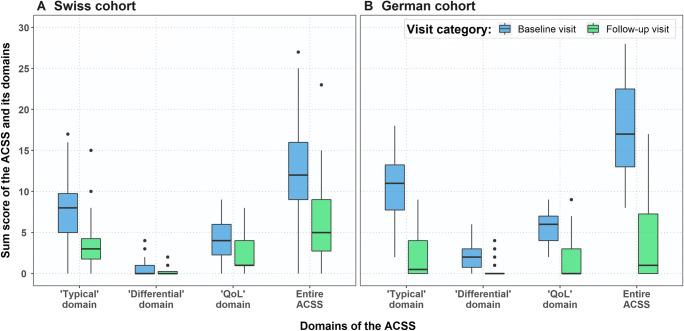
Differences in summary scores of the ACSS and its domains at the baseline and follow-up visits for Swiss and German cohorts

Comparative analysis of the average summary scores of domains and the scores of individual items at baseline and follow-up visits in the Swiss cohort resulted in a statistically significant decrease of the scores after therapy for almost all domains and items of the ACSS as well as the entire ACSS, except for the item on “visible blood in urine” and “Differential” domain (Table 2, Fig. 2A). In the German cohort, differences between scores at baseline and follow-up visits were statistically significant (< 0.05), except for two items of the “Differential” domain: “urethral discharge” and “feeling fever/chills” (Table 2, Fig. 2B).

**Table 2:** Comparison of average (median and interquartile range) scores of the ACSS items and domains at baseline and follow-up visits

Item/domain	Total cohort (*n* = 97)			German cohort (*n* = 26)			Swiss cohort (*n* = 71)		
	Baseline visit	Follow-up visit	P value*	Baseline visit	Follow-up visit	P value*	Baseline visit	Follow-up visit	P value*
*“Typical” domain*									
Urination frequency	2.0 (1.0–3.0)	1.0 (0.0–1.0)	< 0.001	2.0 (1.0–3.0)	0.0 (0.0–1.0)	0.001	1.0 (0.0–2.0)	1.0 (0.0–1.0)	0.026
Urination urgency	2.0 (0.0–3.0)	0.0 (0.0–1.0)	< 0.001	2.0 (1.0–3.0)	0.0 (0.0–0.0)	< 0.001	1.0 (0.0–3.0)	0.0 (0.0–1.0)	0.003
Dysuria	2.0 (0.0–3.0)	0.0 (0.0–1.0)	< 0.001	2.0 (2.0–3.0)	0.0 (0.0–0.0)	< 0.001	2.0 (0.0–3.0)	0.0 (0.0–1.0)	0.007
Incomplete bladder emptying	1.0 (0.0–3.0)	0.0 (0.0–1.0)	< 0.001	2.0 (1.0–3.0)	0.0 (0.0–0.0)	< 0.001	1.0 (0.0–2.0)	0.0 (0.0–1.0)	0.019
Suprapubic pain	2.0 (0.0–3.0)	0.0 (0.0–1.0)	< 0.001	2.0 (1.0–3.0)	0.0 (0.0–1.0)	< 0.001	2.0 (0.0–3.0)	0.0 (0.0–1.0)	0.006
Visible blood in urine	0.0 (0.0–0.0)	0.0 (0.0–0.0)	0.056	0.0 (0.0–1.75)	0.0 (0.0–0.0)	0.015	0.0 (0.0–0.0)	0.0 (0.0–0.0)	0.425
*“Differential” domain*									
Flank pain	0.0 (0.0–1.0)	0.0 (0.0–0.0)	0.065	1.0 (0.0–2.0)	0.0 (0.0–0.0)	0.021	0.0 (0.0–0.0)	0.0 (0.0–0.0)	0.352
Vaginal discharge	0.0 (0.0–0.0)	0.0 (0.0–0.0)	0.040	0.0 (0.0–1.0)	0.0 (0.0–0.0)	0.008	0.0 (0.0–0.0)	0.0 (0.0–0.0)	0.583
Urethral discharge	0.0 (0.0–0.0)	0.0 (0.0–0.0)	0.670	0.0 (0.0–0.0)	0.0 (0.0–0.0)	1.000	0.0 (0.0–0.0)	0.0 (0.0–0.0)	NA
Feeling fever/chills	0.0 (0.0–0.0)	0.0 (0.0–0.0)	0.217	0.0 (0.0–0.0)	0.0 (0.0–0.0)	0.372	0.0 (0.0–0.0)	0.0 (0.0–0.0)	0.347
*“QoL” domain*									
General dyscomfort	2.0 (1.0–2.0)	1.0 (0.0–1.0)	< 0.001	2.0 (1.25–3.0)	0.0 (0.0–1.0)	< 0.001	2.0 (1.0–2.0)	1.0 (1.0–1.0)	0.002
Impact on everyday activity	1.0 (1.0–2.0)	0.0 (0.0–1.0)	< 0.001	2.0 (1.0–2.0)	0.0 (0.0–1.0)	< 0.001	1.0 (1.0–2.0)	0.0 (0.0–1.0)	< 0.001
Impact on social activity	1.0 (1.0–2.0)	0.0 (0.0–1.0)	< 0.001	2.0 (1.0–2.0)	0.0 (0.0–1.0)	< 0.001	1.0 (0.0–2.0)	0.0 (0.0–1.0)	0.002
*Summary scores*									
Typical	8.0 (5.0–12.0)	2.0 (0.0–4.0)	< 0.001	11.0 (8.0–13.75)	0.0 (0.0–4.0)	< 0.001	8.0 (4.5–9.0)	3.0 (1.0–4.0)	< 0.001
Differential	0.0 (0.0–2.0)	0.0 (0.0–0.0)	0.01	2.0 (0.25–2.75)	0.0 (0.0–0.0)	0.005	0.0 (0.0–1.0)	0.0 (0.0–0.0)	0.173
QoL	5.0 (3.0–6.0)	1.0 (0.0–3.5)	< 0.001	5.5 (4.0–7.0)	0.0 (0.0–3.0)	< 0.001	4.0 (2.5–6.0)	1.0 (1.0–3.75)	< 0.001
Entire ACSS	14.0 (10.0–18.0)	4 (1.0–8.5)	< 0.001	17 (13.0–22.0)	1.0 (0.0–8.0)	< 0.001	12.0 (9.0–16.0)	5.0 (2.0–9.0)	< 0.001

*Baseline visit vs. follow-up visit

Median (IQR) difference in summary scores between baseline and follow-up visits of the patients in the Swiss cohort was 6.0 (2.5–8.5) for the “Typical” domain, 0.0 (0.0–1.0) for the “Differential” domain, 2.5 (0.0–4.0) for the “QoL” domain and 9.0 (4.0–14.0) for the entire ACSS score.

The strength of the relationship between the difference in summary scores of the ACSS at baseline and follow-up visits with the “clinical scenario” scale in the Swiss cohort resulted in the following Pearson’s r [95% CI] correlation coefficients: −0.49 [−0.75; −0.08] for “Typical domain; −0.25 [-0.18; 0.60] for “Differential” domain, −0.49 [−0.75; −0.10] for “QoL” domain and − 0.51 [−0.77; −0.11] for the entire ACSS score. As expected, all coefficients were statistically significant (*p* < 0.05), except for the “Differential” domain (*p* = 0.248).

Spearman’s rho between the values of “Dynamics” and the “clinical scenario” was 0.72 (*p* < 0.001) (Suppl. Fig. 4).

### Diagnostic values

**Fig. 3:**
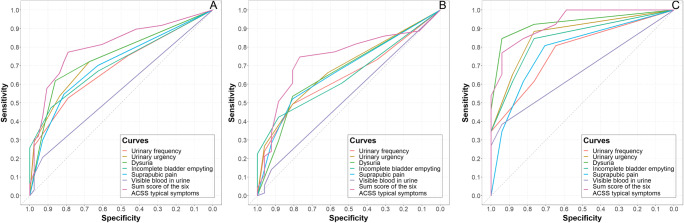
ROC curves for the scores of individual typical symptoms and the summary score of the “Typical” domain of the ACSS in the (**A**) total, (**B**) Swiss and (**C**) German cohorts of recruited patients comparing the results obtained at baseline and follow-up visits

The items and domains of the ACSS demonstrated moderate to good diagnostic values. Figure 3 illustrates the ROC curves for the total cohort (Fig. 3A) and separately for the Swiss (Fig. 3B) and German (Fig. 3C) cohorts.

**Fig. 4:**
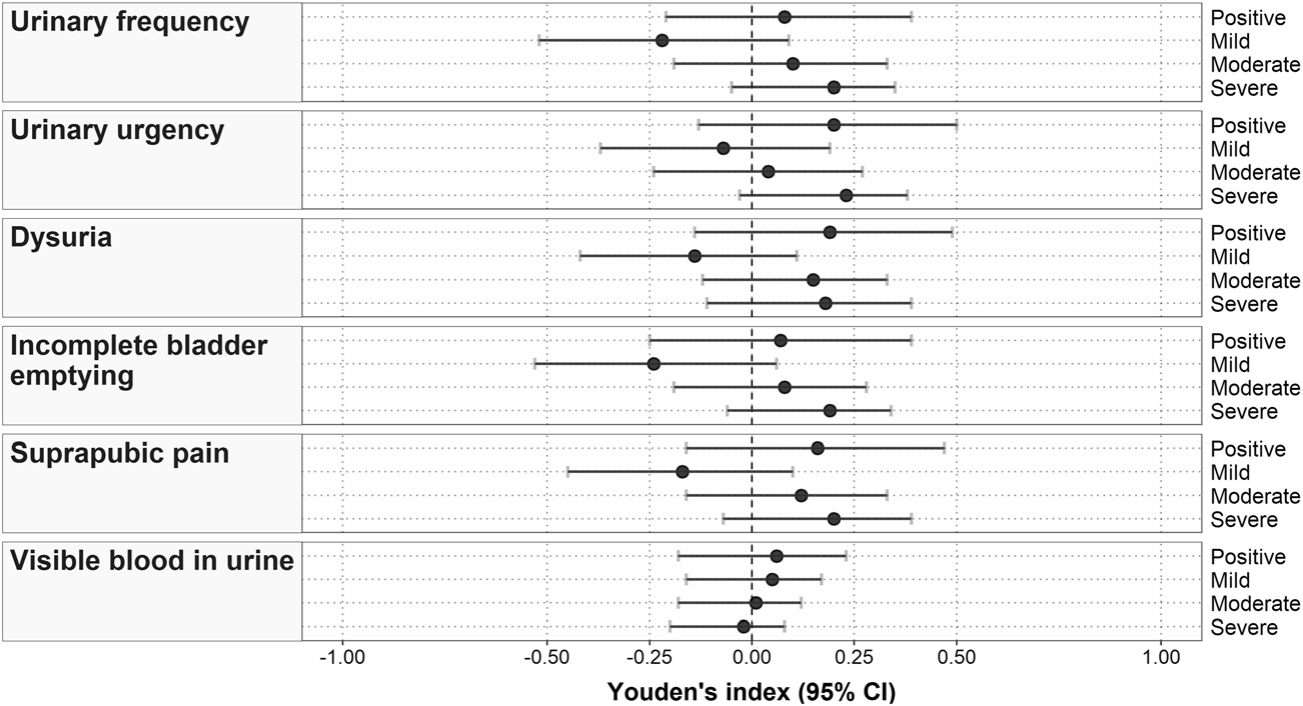
Youden’s index for the ACSS typical symptoms of different severity in the Swiss cohort

Suppl. Table 3 illustrates the average values and 95% confidence intervals for the different diagnostic parameters of the typical symptoms and their severity, according to the respective ACSS items for the Swiss cohort. The optimal values belonged to urinary urgency and dysuria (Suppl. Table 3). The highest values of Youden’s index were obtained for the presence of urinary urgency and dysuria of any severity (Suppl. Table 3, Fig. 4). Concerning symptom intensity the highest values of Youden’s index [95% CI] were found for severe intensities of urinary urgency (0.23 [−0.03; 0.38]), urinary frequency (0.20 [−0.05; 0.35]) and sense of incomplete emptying of the bladder (0.20 [−0.07; 0.39]) (Suppl. Table 3, Fig. 4). The lowest diagnostic value belonged to visible blood in the urine of any intensity (Suppl. Table 3, Figs. 3 and 4).

## Discussion

Our main findings were that the comparative tests showed that the psychometric and discriminative parameters of the German version of the ACSS have similar values for German and Swiss women suffering from AC. The comparative analysis of the internal consistency with the randomly selected reference “International” cohort has shown that the internal consistency of the German ACSS in the Swiss cohort was close to that of the “general population.” Based on extensive statistical evaluations, our study hypothesis was confirmed, and the null hypothesis of inequality of performance of the German ACSS among German-speaking women in both Germany and Switzerland was rejected.

The main scientific and educative activities of ESIU include research on UTI worldwide. Our previous epidemiological trials on cUTI have shown the efficacy of a standardized and unified cross-sectional approach in multinational global studies and have underlined the importance of the exchange of data [35–38]. Unfortunately, at the same time, there is a lack of comprehensive up-to-date information on the global prevalence of uUTI today. Most of the studies about uUTI refer to old and possibly outdated sources, which may not accurately reflect the current epidemiological situation [1, 6].

The diagnostic criteria and treatment recommendations for AC in women have changed during the last decades. Today, AC is considered a benign infection without significant risk of worsening of UTI or serious complications [7]. Since, according to EAU Guidelines urine analyses may lead only to a minimal increase in diagnostic accuracy in patients presenting with typical symptoms of AC, the clinical diagnosis can be made even without a point-of-care urinalysis [7]. The ACSS is a two-part self-reporting questionnaire, which has demonstrated excellent levels of reliability, validity, diagnostic and discriminative abilities in numerous studies [13, 14, 39–49]. It contains ten items on “subjective” and one item on “objective” signs and symptoms. These items are categorized into specific domains, such as “Typical” (urination frequency, urination urgency, painful urination/dysuria, sense of incomplete bladder emptying, suprapubic pain and visible blood in urine), “Differential” (flank pain, abnormal vaginal discharge, discharge from the urethra, feeling fever/high body temperature and measured hyperthermia) and “Quality of Life” (general discomfort, impact on everyday activity and impact on social life). It includes the “Additional” domain containing five questions with dichotomized (“yes/no”) answers on the presence of additional conditions which may affect therapy (menstruations, premenstrual syndrome, signs of menopause, pregnancy and known sugar diabetes). Furthermore, in addition to the four domains mentioned above, the “follow-up” part B of the ACSS includes the “Dynamics” domain to assess the overall clinical outcome reported by the patient. Initially developed in the Uzbek language, the ACSS has been translated and validated in several languages and used as a primary tool for assessment in different countries in different languages [12–14, 39, 43, 47, 48, 50].

The application of the ACSS as a specific and single tool allows to unify the raw data and this may minimize biases. Since, as was recently confirmed, the severity of symptoms plays a major role in the accuracy of the diagnosis rather than the presence or absence of symptoms, and ACSS makes it possible to analyze the prevalence of AC and its symptoms of different severity. Thus, personal contacts, in such symptomatic and benign diseases as AC, could be minimized by using telephone-based evaluation and standardized treatment algorithms [51].

With its high values for reliability and diagnostic capabilities, the ACSS has been recommended as a valuable tool for diagnosis and patient-related outcome in female patients with AC by the National Guidelines of two European countries: the German interdisciplinary S3 Guidelines (2017) and National Clinical Recommendations of the Russian Federation (2019) [11, 52].

Besides, with the help of the ACSS, evaluation and tentative diagnosis of AC can be made by medical assistants involving a certified physician only for confirmation of the diagnosis and prescription of the recommended treatment modality. Implementation of self-reporting questionnaires like the ACSS in the routine may have great advantages especially in the current times of the worldwide COVID-19 pandemic, dictating restriction of personal contacts to the essential minimum and avoidance of places where people are at risk of infection including doctors’ offices, unless strictly necessary [53].

The GPIU.COM-Study aimed to highlight contemporary aspects of uUTI in women, including but not limited to the epidemiology of uUTI, the prevalence of risk factors, the most common causative uropathogens and their resistance to commonly prescribed antimicrobial agents, etc. This part of the GPIU.COM study used the ACSS as the primary tool for the diagnosis and patient-reported outcome measurement in female patients with AC [12].

Strong relationships between the physician’s independent assessment of the changes in the patient’s condition using the leveled values of “clinical scenario” and the differences in summary scores of the “Typical” and “QoL” domains of the ACSS between the baseline and follow-up visits showed the high responsiveness of these summary scores to changes. The consistency of the scores of the “Dynamics” domain with the physician’s independent assessment of the changes in the patient’s condition, vouches for the validity of the “Dynamics” domain. These results testify to the desirability of using the ACSS in continuous monitoring of the progress in the patient’s well-being during the symptomatic course of AC, which suggests the feasibility of using the ACSS not only as a diagnostic tool but also as a valuable patient-reported outcome measure (PROM).

However, when analyzing clinical parameters and diagnostic criteria of the patients, we found discrepancies in sensitivity and specificity between the two cohorts. Some discrepancies in clinical parameters and diagnostic criteria between Swiss and German cohorts might be the result of multiple factors, which we have found reasonable to highlight below.

One possible reason is the difference in recruiting patients due to different definitions and diagnostic standards in the German and Swiss Guidelines on Urological Infections [10, 11, 54]. According to the German Guideline the ACSS is recommended for the diagnosis of AC in female patients [11]. Therefore, the recruitment of the patients in the German cohort was primarily based on the cut-off point of the ACSS questionnaire, whereas the patients of the Swiss cohort were probably recruited according to the definition of AC in the Guidelines of the Swiss Society for Infectious Diseases, which implies the presence of symptoms AND pyuria [10]. Second, although homogeneous in the main demographic characteristics, patients of the two cohorts may have differed in clinical variables, such as the results of urine tests and summary scores of the ACSS domains (Table 1). These parameters could serve as co-factors and lead to selection biases during recruitment between the study cohorts, as observed and reported earlier [50]. Such dissimilarity of recommendations may affect the study results and clinical practice [44, 46].

The main limitation of the current study is the discrepancies found in diagnostic values of the symptoms between the two cohorts, which may be explained best by minor differences between the diagnostic criteria recommended in different National Guidelines. Nevertheless, our results support that the ACSS may serve as a single point-of-care test in future studies.

Another limitation may be the non-interventional character of the study, which could not guarantee that follow-up visits were performed by all patients.

## Conclusions

Our study confirmed the equality of performance of the German ACSS among German-speaking women both in Germany and Switzerland based on the reliability, validity and diagnostic values of the ACSS.

The GPIU.COM-Study should be enlarged to a wider cohort of patients with AC to be monitored using the ACSS as both a primary diagnostic tool and PROM, respectively. Healthy female subjects (negative controls) and patients with urological disorders other than AC (positive controls) should also be included for further studies on the diagnostic accuracy of the ACSS. By using a telephone-based algorithm for diagnosis and treatment of AC the ACSS might become a cost-effective aid for physicians and patients alike.

## Supplementary Information

Supplementary Table 1. Internal consistency (Cronbach’s alpha coefficient) of different domains of the German version of the ACSS. 2A: Comparison between Swiss and German cohorts of the study. 2B: comparison between Swiss and reference “International” cohort, randomly created from the e-USQOLAT database. (DOC 34 kb)

Supplementary Table 2. Strength of associations (Pearson’s correlation coefficient and 95% confidence intervals) between the domains of the ACSS and the validated German version of the EQ5-5D-3L questionnaire. (DOC 46 kb)

Supplementary Table 3. Average diagnostic values and 95% confidence intervals for symptoms of the “Typical” domain of the ACSS and different levels of their severity in the Swiss cohort. (DOC 65 kb)

Supplementary Fig. 1. German version of the Acute Cystitis Symptom Score used in the study. (PDF 620 kb)

Supplementary Fig. 2. Flowchart of the creation of the reference “International” cohort. (Note: pre-randomization inclusion criteria were applied to create a sample homogeneous with the Swiss cohort.) (PNG 324 kb)

Supplementary Fig. 3. (A-D) Relationships between the items of the validated German 3-level version of the EuroQoL-5 Dimension (EQ-5D-3L) Health Questionnaire and summary scores of: A: “Typical” domain, B: “Differential” domain, C: “QoL” domain of the ACSS and D: entire ACSS. (PNG 188 kb)

(PNG 161 kb)

(PNG 175 kb)

(PNG 135 kb)

Supplementary Fig. 4. Relationships between the items of the “Dynamics” domain of the ACSS and the “clinical scenario” assessed by the physician. (PNG 82 kb)

## Footnotes

1 The median (IQR) number of patients recruited between February 27, 2016, and January 30, 2020, was 18 (11.0–26.0) per institution (range 11–31).

## Abbreviations

AC: acute cystitis
ACSS: the Acute Cystitis Symptom Score
AUC: urea under the ROC curve
CI: confidence intervals
COVID-19: coronavirus disease of 2019
cUTI: complicated urinary tract infections
EAU: European Association of Urology
EMA: European Medicines Agency
EQ-5D-3L: three-level version of the EuroQoL-5 Dimension Health Questionnaire
ESIU: European Section for Infections in Urology
FDA: the United States Food and Drug Administration
GPIU.COM: Global Prevalence Study of Infections in Urinary tract in a Community setting
IQR: interquartile range
PROM: patient-reported outcome measure
ROC: receiver operating characteristic
UTI: urinary tract infections
uUTI: uncomplicated urinary tract infections
WHO: World Health Organization
