# Supplementary material for: Linguistic and clinical validation of the acute cystitis symptom score in German-speaking Swiss women with acute cystitis
Source: Int Urogynecol J. 2021 Jun 25;32(12):3275–86. doi: 10.1007/s00192-021-04864-1 (PMC8227360; doi:10.1007/s00192-021-04864-1)
Supplement: Supplementary file 1 — Internal consistency (Cronbach’s alpha coefficient) of different domains of the German version of the ACSS. 2A: Comparison between Swiss and German cohorts of the study. 2B: comparison between Swiss and reference “International” cohort, randomly created from the e-USQOLAT database. (DOC 34 kb) [file 192_2021_4864_MOESM1_ESM.doc]

| **Domain of the ACSS** | **Total cohort [95%CI]** | **German cohort [95%CI]** | **Swiss cohort [95%CI]** | **P-value*** |
| --- | --- | --- | --- | --- |
| **Typical** | 0.82 [0.78; 0.87] | 0.89 [0.82; 0.93] | 0.79 [0.72; 0.85] | 0.07 |
| **Differential** | 0.32 [0.15; 0.50] | 0.38 [0.01; 0.63] | 0.18 [-0.12; 0.42] | 0.44 |
| **QoL** | 0.91 [0.89; 0.94] | 0.93 [0.89; 0.96] | 0.91 [0.87; 0.93] | 0.52 |
| **Entire ACSS** | 0.86 [0.83; 0.89] | 0.90 [0.85; 0.94] | 0.85 [0.79; 0.89] | 0.15 |
| *Swiss cohort vs. German cohort. | |  |  |  |

| **Domain of the ACSS** | **Total cohort [95%CI]** | **Swiss cohort [95%CI]** | **International cohort [95%CI]** | **P-value*** |
| --- | --- | --- | --- | --- |
| **Typical** | 0.84 [0.81; 0.87] | 0.79 [0.72; 0.85] | 0.86 [0.82; 0.90] | 0.08 |
| **Differential** | 0.37 [0.26; 0.49] | 0.18 [-0.12; 0.42] | 0.43 [0.23; 0.58] | 0.17 |
| **QoL** | 0.91 [0.89; 0.93] | 0.91 [0.87; 0.93] | 0.91 [0.88; 0.94] | 0.65 |
| **Entire ACSS** | 0.87 [0.85; 0.89] | 0.85 [0.79; 0.89] | 0.89 [0.85; 0.92] | 0.12 |
| *Swiss cohort vs. International reference cohort. | | | | |
