# Supplementary material for: Linguistic and clinical validation of the acute cystitis symptom score in German-speaking Swiss women with acute cystitis
Source: Int Urogynecol J. 2021 Jun 25;32(12):3275–86. doi: 10.1007/s00192-021-04864-1 (PMC8227360; doi:10.1007/s00192-021-04864-1)
Supplement: Supplementary file 2 — Strength of associations (Pearson’s correlation coefficient and 95% confidence intervals) between the domains of the ACSS and the validated German version of the EQ5-5D-3L questionnaire. (DOC 46 kb) [file 192_2021_4864_MOESM2_ESM.doc]

| **EQ-5D-3L Questionnaire domains** | **ACSS domains** | | | |
| --- | --- | --- | --- | --- |
|  | **Typical domain** | **Differential** | **QoL** | **Entire ACSS** |
| *Total cohort* |  |  |  |  |
| Mobility | 0.29 [0.12; 0.45] *** | 0.07 [-0.11; 0.25] N.S. | 0.41 [0.25; 0.55] *** | 0.36 [0.19; 0.51] *** |
| Self-care | 0.21 [0.03; 0.38] * | -0.05 [-0.22; 0.13] N.S. | 0.25 [0.08; 0.41] ** | 0.23 [0.05; 0.4] * |
| Usual activities | 0.23 [0.05; 0.4] * | -0.03 [-0.21; 0.15] N.S. | 0.39 [0.23; 0.53] *** | 0.28 [0.11; 0.44] ** |
| Pain/Discomfort | 0.38 [0.21; 0.52] *** | 0.12 [-0.06; 0.29] N.S. | 0.39 [0.23; 0.54] *** | 0.42 [0.26; 0.56] *** |
| Anxiety/Depression | 0.16 [-0.02; 0.33] N.S. | 0.03 [-0.15; 0.21] N.S. | 0.28 [0.11; 0.44] ** | 0.2 [0.02; 0.37] * |
| General health status | -0.42 [-0.56; -0.25] *** | -0.26 [-0.42; -0.09] ** | -0.56 [-0.67; -0.42] *** | -0.5 [-0.63; -0.34] *** |
| *German cohort* |  |  |  |  |
| Mobility | 0.3 [-0.04; 0.58] N.S. | -0.15 [-0.46; 0.19] N.S. | 0.29 [-0.05; 0.56] N.S. | 0.28 [-0.07; 0.57] N.S. |
| Self-care | 0.18 [-0.16; 0.49] N.S. | -0.18 [-0.48; 0.16] N.S. | 0.19 [-0.15; 0.48] N.S. | 0.16 [-0.19; 0.48] N.S. |
| Usual activities | 0.19 [-0.16; 0.5] N.S. | -0.23 [-0.52; 0.11] N.S. | 0.19 [-0.15; 0.49] N.S. | 0.16 [-0.19; 0.48] N.S. |
| Pain/Discomfort | 0.34 [0.01; 0.61] * | 0.1 [-0.25; 0.42] N.S. | 0.35 [0.02; 0.61] * | 0.4 [0.06; 0.65] * |
| Anxiety/Depression | 0.39 [0.06; 0.65] * | -0.12 [-0.43; 0.22] N.S. | 0.31 [-0.02; 0.58] N.S. | 0.32 [-0.02; 0.6] N.S. |
| General health status | -0.59 [-0.78; -0.32] *** | -0.37 [-0.63; -0.04] * | -0.78 [-0.88; -0.6] *** | -0.69 [-0.84; -0.45] *** |
| *Swiss cohort* |  |  |  |  |
| Mobility | 0.29 [0.07; 0.47] ** | 0.26 [0.05; 0.44] * | 0.48 [0.3; 0.63] *** | 0.41 [0.21; 0.57] *** |
| Self-care | 0.23 [0.02; 0.43] * | 0.06 [-0.16; 0.26] N.S. | 0.31 [0.11; 0.49] ** | 0.29 [0.07; 0.47] ** |
| Usual activities | 0.26 [0.05; 0.45] * | 0.17 [-0.04; 0.37] N.S. | 0.51 [0.34; 0.65] *** | 0.37 [0.17; 0.55] *** |
| Pain/Discomfort | 0.4 [0.21; 0.57] *** | 0.18 [-0.03; 0.38] N.S. | 0.42 [0.23; 0.58] *** | 0.45 [0.26; 0.61] *** |
| Anxiety/Depression | 0.04 [-0.18; 0.26] N.S. | 0.13 [-0.08; 0.34] N.S. | 0.27 [0.06; 0.46] * | 0.14 [-0.08; 0.35] N.S. |
| General health status | -0.33 [-0.52; -0.12] ** | -0.12 [-0.32; 0.1] N.S. | -0.44 [-0.6; -0.25] *** | -0.39 [-0.57; -0.19] *** |
| *Note: *** - p<0.001, ** - p<0.01, * - p<0.05, N.S. - p≥0.05* | | | | |
