# Supplementary material for: Linguistic and clinical validation of the acute cystitis symptom score in German-speaking Swiss women with acute cystitis
Source: Int Urogynecol J. 2021 Jun 25;32(12):3275–86. doi: 10.1007/s00192-021-04864-1 (PMC8227360; doi:10.1007/s00192-021-04864-1)
Supplement: Supplementary file 3 — Average diagnostic values and 95% confidence intervals for symptoms of the “Typical” domain of the ACSS and different levels of their severity in the Swiss cohort. (DOC 65 kb) [file 192_2021_4864_MOESM3_ESM.doc]

| **Symptom** | **Sensitivity** | **Specificity** | **Positive predictive value** | **Negative predictive value** | **Diagnostic odds ratio** | **Youden's index** | **Area under the ROC-curve** |
| --- | --- | --- | --- | --- | --- | --- | --- |
| **Urinary frequency** | 0.73 [0.61; 0.83] | 0.35 [0.17; 0.56] | 0.75 [0.64; 0.85] | 0.32 [0.16; 0.52] | 1.45 [0.55; 3.80] | 0.08 [-0.21; 0.39] | 0.54 [0.44; 0.64] |
| Mild | 0.24 [0.15; 0.36] | 0.54 [0.33; 0.73] | 0.59 [0.39; 0.76] | 0.21 [0.12; 0.32] | 0.37 [0.14; 0.94] | -0.22 [-0.52; 0.09] | 0.40 [0.29; 0.50] |
| Moderate | 0.25 [0.16; 0.37] | 0.85 [0.65; 0.96] | 0.82 [0.60; 0.95] | 0.29 [0.19; 0.41] | 1.87 [0.57; 6.15] | 0.10 [-0.19; 0.33] | 0.56 [0.46; 0.65] |
| Severe | 0.24 [0.15; 0.36] | 0.96 [0.80; 0.99] | 0.94 [0.73; 0.99] | 0.32 [0.22; 0.43] | 7.87 [0.99; 62.48] | 0.20 [-0.05; 0.35] | 0.63 [0.56; 0.71] |
| **Urinary urgency** | 0.66 [0.54; 0.77] | 0.54 [0.33; 0.73] | 0.80 [0.67; 0.89] | 0.37 [0.22; 0.54] | 2.28 [0.92; 5.70] | 0.20 [-0.13; 0.50] | 0.58 [0.49; 0.68] |
| Mild | 0.20 [0.11; 0.31] | 0.73 [0.52; 0.88] | 0.67 [0.43; 0.85] | 0.25 [0.16; 0.36] | 0.67 [0.23; 1.90] | -0.07 [-0.37; 0.19] | 0.46 [0.34; 0.57] |
| Moderate | 0.20 [0.11; 0.31] | 0.85 [0.65; 0.96] | 0.78 [0.52; 0.94] | 0.28 [0.18; 0.39] | 1.35 [0.40; 4.55] | 0.04 [-0.24; 0.27] | 0.53 [0.42; 0.64] |
| Severe | 0.27 [0.17; 0.39] | 0.96 [0.80; 0.99] | 0.95 [0.75; 0.99] | 0.32 [0.22; 0.44] | 9.13 [1.16; 72.15] | 0.23 [-0.03; 0.38] | 0.64 [0.57; 0.71] |
| **Dysuria** | 0.65 [0.53; 0.76] | 0.54 [0.33; 0.73] | 0.79 [0.67; 0.89] | 0.36 [0.21; 0.53] | 2.15 [0.86; 5.34] | 0.19 [-0.14; 0.49] | 0.58 [0.48; 0.67] |
| Mild | 0.13 [0.06; 0.23] | 0.73 [0.52; 0.88] | 0.56 [0.30; 0.80] | 0.23 [0.15; 0.34] | 0.39 [0.13; 1.20] | -0.14 [-0.42; 0.11] | 0.40 [0.26; 0.53] |
| Moderate | 0.23 [0.13; 0.34] | 0.92 [0.75; 0.99] | 0.89 [0.65; 0.99] | 0.30 [0.21; 0.42] | 3.49 [0.74; 16.38] | 0.15 [-0.12; 0.33] | 0.60 [0.51; 0.69] |
| Severe | 0.30 [0.19; 0.42] | 0.88 [0.70; 0.98] | 0.88 [0.68; 0.97] | 0.32 [0.21; 0.43] | 3.22 [0.87; 11.89] | 0.18 [-0.11; 0.39] | 0.60 [0.51; 0.68] |
| **Suprapubic pain** | 0.61 [0.48; 0.72] | 0.46 [0.27; 0.67] | 0.75 [0.62; 0.86] | 0.30 [0.17; 0.47] | 1.32 [0.53; 3.26] | 0.07 [-0.25; 0.39] | 0.53 [0.44; 0.62] |
| Mild | 0.18 [0.10; 0.29] | 0.58 [0.37; 0.77] | 0.54 [0.33; 0.74] | 0.21 [0.12; 0.32] | 0.31 [0.11; 0.82] | -0.24 [-0.53; 0.06] | 0.37 [0.26; 0.49] |
| Moderate | 0.20 [0.11; 0.31] | 0.88 [0.70; 0.98] | 0.82 [0.57; 0.96] | 0.29 [0.19; 0.40] | 1.88 [0.49; 7.17] | 0.08 [-0.19; 0.28] | 0.56 [0.45; 0.66] |
| Severe | 0.23 [0.13; 0.34] | 0.96 [0.81; 0.99] | 0.94 [0.71; 0.99] | 0.32 [0.22; 0.43] | 7.56 [0.95; 60.15] | 0.19 [-0.06; 0.34] | 0.66 [0.61; 0.71] |
| **Sense of incomplete bladder emptying** | 0.66 [0.54; 0.77] | 0.50 [0.30; 0.70] | 0.78 [0.66; 0.88] | 0.35 [0.20; 0.53] | 1.96 [0.79; 4.88] | 0.16 [-0.16; 0.47] | 0.57 [0.47; 0.66] |
| Mild | 0.14 [0.07; 0.24] | 0.69 [0.48; 0.86] | 0.56 [0.31; 0.78] | 0.23 [0.14; 0.34] | 0.37 [0.13; 1.07] | -0.17 [-0.45; 0.1] | 0.39 [0.26; 0.52] |
| Moderate | 0.24 [0.15; 0.36] | 0.88 [0.70; 0.98] | 0.85 [0.62; 0.97] | 0.30 [0.20; 0.41] | 2.41 [0.64; 9.04] | 0.12 [-0.16; 0.33] | 0.57 [0.48; 0.67] |
| Severe | 0.28 [0.18; 0.40] | 0.92 [0.75; 0.99] | 0.91 [0.71; 0.99] | 0.32 [0.22; 0.44] | 4.71 [1.02; 21.78] | 0.20 [-0.07; 0.39] | 0.61 [0.53; 0.70] |
| **Visible blood in the urine** | 0.14 [0.07; 0.24] | 0.92 [0.75; 0.99] | 0.83 [0.52; 0.98] | 0.28 [0.19; 0.39] | 1.97 [0.40; 9.65] | 0.06 [-0.18; 0.23] | 0.56 [0.44; 0.68] |
| Mild | 0.08 [0.03; 0.17] | 0.96 [0.80; 0.99] | 0.86 [0.42; 0.99] | 0.28 [0.19; 0.38] | 2.31 [0.26; 20.14] | 0.05 [-0.16; 0.17] | 0.57 [0.42; 0.71] |
| Moderate | 0.04 [0.01; 0.12] | 0.96 [0.81; 0.99] | 0.75 [0.19; 0.99] | 0.28 [0.19; 0.38] | 1.15 [0.11; 11.53] | 0.01 [-0.18; 0.12] | 0.64 [0.59; 0.68] |
| Severe | 0.01 [0.01; 0.08] | 0.96 [0.8; 0.99] | 0.50 [0.01; 0.99] | 0.26 [0.18; 0.36] | 0.36 [0.02; 5.93] | -0.02 [-0.2; 0.08] | 0.62 [0.13; 0.99] |
