# Supplementary material for: Linguistic and clinical validation of the acute cystitis symptom score in German-speaking Swiss women with acute cystitis
Source: Int Urogynecol J. 2021 Jun 25;32(12):3275–86. doi: 10.1007/s00192-021-04864-1 (PMC8227360; doi:10.1007/s00192-021-04864-1)
Supplement: Supplementary file 4 — German version of the Acute Cystitis Symptom Score used in the study. (PDF 620 kb) [file 192_2021_4864_MOESM4_ESM.pdf]

ACSS-Fragebogen

Erstvorstellung (Diagnose) – Teil A

Uhrzeit: ____UU:____MM Datum der Untersuchung: / / (Tag/Monat/Jahr)

Bitte geben Sie an, ob Sie unten genannte Symptome innerhalb der letzten 24 Stunden bemerkt haben, und bewerten Sie bitte deren Intensität *(nur eine Antwort für jedes einzelne Symptom)*

			0	1	2	3
**Typische Symptome**	1	Häufiges Wasserlassen mit geringen Urinportionen *(wiederholte WC-Besuche)*	☐ Nein<br>*bis 4-mal täglich*	☐ Ja, wenig<br>*5-6-mal täglich*	☐ Ja, mäßig<br>*7-8-mal täglich*	☐ Ja, stark<br>*9-10-mal täglich*
	2	Starker, unwillkürlicher Harndrang	☐ Nein	☐ Ja, wenig	☐ Ja, mäßig	☐ Ja, stark
	3	Schmerzen und Brennen beim Wasserlassen	☐ Nein	☐ Ja, wenig	☐ Ja, mäßig	☐ Ja, stark
	4	Gefühl einer unvollständigen Harnblasenentleerung	☐ Nein	☐ Ja, wenig	☐ Ja, mäßig	☐ Ja, stark
	5	Schmerzen oder Beschwerden *(unangenehmes Druckgefühl)* im Unterbauch oder Beckenbereich	☐ Nein	☐ Ja, wenig	☐ Ja, mäßig	☐ Ja, stark
	6	Sichtbares Blut im Urin	☐ Nein	☐ Ja, wenig	☐ Ja, mäßig	☐ Ja, stark
					**Gesamtpunktzahl =** ☐	**Punkte**
**Differentialdiagnose**	7	Schmerzen in der Lendengegend *(Flanke)* *(oft einseitig - auf einer Seite)*	☐ Nein	☐ Ja, wenig	☐ Ja, mäßig	☐ Ja, stark
	8	Neuer oder zunehmender Ausfluss aus der Scheide	☐ Nein	☐ Ja, wenig	☐ Ja, mäßig	☐ Ja, stark
	9	Eitriger Ausfluss aus der Harnröhre *(unabhängig vom Wasserlassen)*	☐ Nein	☐ Ja, wenig	☐ Ja, mäßig	☐ Ja, stark
	10	Erhöhte Körpertemperatur *(über 37,5°C)* / Schüttelfrost	☐ Nein	☐ Ja, wenig	☐ Ja, mäßig	☐ Ja, stark
		Wenn Sie Temperatur gemessen haben, geben Sie diese bitte an	≤37,5 ˚C	37,6-37,9 ˚C	38,0-38,9 ˚C	≥39,0 ˚C
					**Gesamtpunktzahl =** ☐	**Punkte**

Lebensqualität

11 **Bitte geben Sie an, wie stark ausgeprägt die durch die oben genannten Symptome hervorgerufenen Beschwerden innerhalb der letzten 24 Stunden waren *(wählen Sie bitte nur eine am ehesten zutreffende Antwort)*:**

- ☐ 0 Keine Beschwerden *(keine Symptome, fühle mich wie immer)*
- ☐ 1 Geringe Beschwerden *(fühle mich etwas unwohler als sonst)*
- ☐ 2 Starke Beschwerden *(fühle mich merklich schlechter als sonst)*
- ☐ 3 Sehr starke Beschwerden *(fühle mich schrecklich)*

12 **Bitte geben Sie an, wie weit die oben genannten Symptome Ihre alltägliche Aktivität / Leistungsfähigkeit innerhalb der letzten 24 Stunden beeinträchtigt haben *(wählen Sie bitte nur eine am ehesten zutreffende Antwort)*:**

- ☐ 0 Überhaupt nicht beeinträchtigt *(arbeite wie an gewöhnlichen Tagen, ohne Beschwerden)*
- ☐ 1 Ein wenig beeinträchtigt *(wegen der Symptome arbeite ich etwas weniger)*
- ☐ 2 Bedeutend beeinträchtigt *(alltägliche Arbeit ist anstrengend geworden)*
- ☐ 3 Stark beeinträchtigt *(ich kann praktisch nicht arbeiten)*

13 **Bitte geben Sie an, wie weit die oben genannten Symptome Ihre gesellschaftlichen Aktivitäten (Besuche machen, sich mit Freunden treffen usw.) innerhalb der letzten 24 Stunden beeinträchtigt haben *(wählen Sie bitte nur eine am ehesten zutreffende Antwort)*:**

- ☐ 0 Überhaupt nicht beeinträchtigt *(es hat sich nichts geändert, ich lebe so wie vorher)*
- ☐ 1 Ein wenig beeinträchtigt *(eine geringe Reduzierung der Aktivität)*
- ☐ 2 Bedeutend beeinträchtigt *(viel weniger aktiv, bleibe mehr zu Hause)*
- ☐ 3 Sehr stark beeinträchtigt *(schrecklich, kann das Haus praktisch nicht verlassen)*

Gesamtpunktzahl = ☐ **Punkte**

Begleitumstände

14 **Bitte geben Sie an, ob zum Zeitpunkt des Ausfüllens des Fragenbogens bei Ihnen folgendes zutrifft:**

Menstruation *(Regel)* ?	☐ Nein	☐ Ja
Prämenstruelle Beschwerden *(Beschwerden in der Zeit vor der Regel)* ?	☐ Nein	☐ Ja
Klimakterisches Syndrom *(Beschwerden in den Wechseljahren)* ?	☐ Nein	☐ Ja
Schwangerschaft?	☐ Nein	☐ Ja
Zuckerkrankheit?	☐ Nein	☐ Ja

HALT

Bitte vergessen Sie nicht, den ausgefüllten Fragebogen Ihrem Arzt zurückzugeben.

Wir danken Ihnen für Ihre Mitarbeit!

Copyrighted. Free for personal use only.
For research and economical purposes, please contact the copyright holders.

Acute Cystitis Symptom Score (ACSS) © 2015. http://inter.interoco.com/copyright-depository/work-of-science/1013-1951954939.html

ACSS-Fragebogen

Kontrollvorstellung (Folgebefund) – Teil B

Uhrzeit: ____UU:____MM Datum der Untersuchung: / / (Tag/Monat/Jahr)

Bitte geben Sie an, ob Sie irgendwelche Änderungen in Ihrem Zustand seit dem Ausfüllen des ersten Teils unseres Fragebogens bemerkt haben? *(kreuzen Sie bitte Ihre Antwort an)*:

Dynamik

- ☐ 0 Ich fühle mich jetzt ausgezeichnet *(alle Symptome sind endgültig vergangen)*
- ☐ 1 Mir geht es jetzt wesentlich besser *(die Mehrheit der Symptome sind vergangen)*
- ☐ 2 Ich fühle mich jetzt nur gering besser *(die Mehrheit der Symptome sind immer noch da)*
- ☐ 3 Es gibt jetzt keine Änderung meines Zustands *(alle Symptome sind noch vorhanden)*
- ☐ 4 Es ist jetzt schlimmer geworden *(mein Zustand hat sich verschlechtert)*

Bitte geben Sie an, ob Sie unten genannte Symptome innerhalb der letzten 24 Stunden bemerkt haben, und bewerten Sie bitte deren Intensität *(nur <u>eine</u> Antwort für jedes einzelne Symptom)*:

			0	1	2	3
Typische Symptome	1	Häufiges Wasserlassen mit geringen Urinportionen *(wiederholte WC-Besuche)*	☐ Nein<br>*bis 4-mal täglich*	☐ Ja, wenig<br>*5-6-mal täglich*	☐ Ja, mäßig<br>*7-8-mal täglich*	☐ Ja, stark<br>*9-10-mal täglich*
	2	Starker, unwillkürlicher Harndrang	☐ Nein	☐ Ja, wenig	☐ Ja, mäßig	☐ Ja, stark
	3	Schmerzen und Brennen beim Wasserlassen	☐ Nein	☐ Ja, wenig	☐ Ja, mäßig	☐ Ja, stark
	4	Gefühl einer unvollständigen Harnblasenentleerung	☐ Nein	☐ Ja, wenig	☐ Ja, mäßig	☐ Ja, stark
	5	Schmerzen oder Beschwerden *(unangenehmes Druckgefühl)* im Unterbauch oder Beckenbereich	☐ Nein	☐ Ja, wenig	☐ Ja, mäßig	☐ Ja, stark
	6	Sichtbares Blut im Urin	☐ Nein	☐ Ja, wenig	☐ Ja, mäßig	☐ Ja, stark
					**Gesamtpunktzahl =**	**Punkte**
Differentialdiagnose	7	Schmerzen in der Lendengegend *(Flanke)* *(oft einseitig - auf einer Seite)*	☐ Nein	☐ Ja, wenig	☐ Ja, mäßig	☐ Ja, stark
	8	Neuer oder zunehmender Ausfluss aus der Scheide	☐ Nein	☐ Ja, wenig	☐ Ja, mäßig	☐ Ja, stark
	9	Eitriger Ausfluss aus der Harnröhre *(unabhängig vom Wasserlassen)*	☐ Nein	☐ Ja, wenig	☐ Ja, mäßig	☐ Ja, stark
	10	Erhöhte Körpertemperatur *(über 37,5°C)* / Schüttelfrost	☐ Nein	☐ Ja, wenig	☐ Ja, mäßig	☐ Ja, stark
		Wenn Sie Temperatur gemessen haben, geben Sie diese bitte an	≤37,5 ˚C	37,6-37,9 ˚C	38,0-38,9 ˚C	≥39,0 ˚C
					**Gesamtpunktzahl =**	**Punkte**

Lebensqualität

11 **Bitte geben Sie an, wie stark ausgeprägt die durch die oben genannten Symptome hervorgerufenen Beschwerden innerhalb der letzten 24 Stunden waren *(wählen Sie bitte nur <u>eine</u> am ehesten zutreffende Antwort)*:**

- ☐ 0 Keine Beschwerden *(keine Symptome, fühle mich wie immer)*
- ☐ 1 Geringe Beschwerden *(fühle mich etwas unwohler als sonst)*
- ☐ 2 Starke Beschwerden *(fühle mich merklich schlechter als sonst)*
- ☐ 3 Sehr starke Beschwerden *(fühle mich schrecklich)*

12 **Bitte geben Sie an, wie weit die oben genannten Symptome Ihre alltägliche Aktivität / Leistungsfähigkeit innerhalb der letzten 24 Stunden beeinträchtigt haben *(wählen Sie bitte nur <u>eine</u> am ehesten zutreffende Antwort)*:**

- ☐ 0 Überhaupt nicht beeinträchtigt *(arbeite wie an gewöhnlichen Tagen, ohne Beschwerden)*
- ☐ 1 Ein wenig beeinträchtigt *(wegen der Symptome arbeite ich etwas weniger)*
- ☐ 2 Bedeutend beeinträchtigt *(alltägliche Arbeit ist anstrengend geworden)*
- ☐ 3 Stark beeinträchtigt *(ich kann praktisch nicht arbeiten)*

13 **Bitte geben Sie an, wie weit die oben genannten Symptome Ihre gesellschaftlichen Aktivitäten (Besuche machen, sich mit Freunden treffen usw.) innerhalb der letzten 24 Stunden beeinträchtigt haben *(wählen Sie bitte nur <u>eine</u> am ehesten zutreffende Antwort)*:**

- ☐ 0 Überhaupt nicht beeinträchtigt *(es hat sich nichts geändert, ich lebe so wie vorher)*
- ☐ 1 Ein wenig beeinträchtigt *(eine geringe Reduzierung der Aktivität)*
- ☐ 2 Bedeutend beeinträchtigt *(viel weniger aktiv, bleibe mehr zu Hause)*
- ☐ 3 Sehr stark beeinträchtigt *(schrecklich, kann das Haus praktisch nicht verlassen)*

Gesamtpunktzahl = Punkte

Begleitumstände

14 **Bitte geben Sie an, ob zum Zeitpunkt des Ausfüllens des Fragenbogens bei Ihnen folgendes zutrifft:**

Menstruation *(Regel)*?	☐ Nein	☐ Ja
Prämenstruelle Beschwerden *(Beschwerden in der Zeit vor der Regel)*?	☐ Nein	☐ Ja
Klimakterisches Syndrom *(Beschwerden in den Wechseljahren)*?	☐ Nein	☐ Ja
Schwangerschaft?	☐ Nein	☐ Ja
Zuckerkrankheit?	☐ Nein	☐ Ja

HALT

Bitte vergessen Sie nicht, den ausgefüllten Fragebogen Ihrem Arzt zurückzugeben.

Wir danken Ihnen für Ihre Mitarbeit!

Copyrighted. Free for personal use only.
For research and economical purposes, please contact the copyright holders.

Acute Cystitis Symptom Score (ACSS) © 2015. http://inter.interoco.com/copyright-depository/work-of-science/1013-1951954939.html
