# Supplementary figures and images for: Linguistic and clinical validation of the acute cystitis symptom score in German-speaking Swiss women with acute cystitis
Source: Int Urogynecol J. 2021 Jun 25;32(12):3275–86. doi: 10.1007/s00192-021-04864-1 (PMC8227360; doi:10.1007/s00192-021-04864-1)

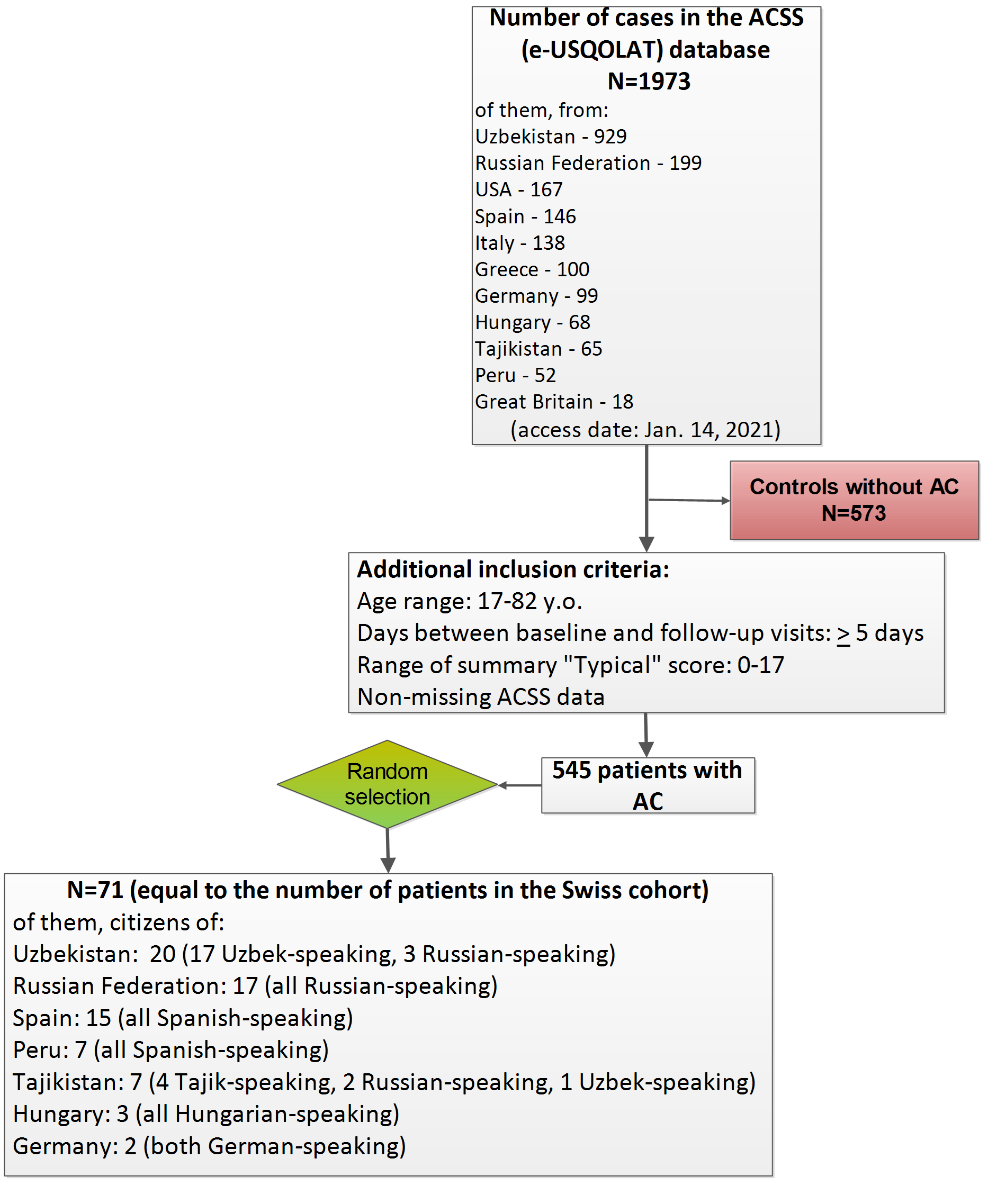

Supplement: Supplementary file 5 — Flowchart of the creation of the reference “International” cohort. (Note: pre-randomization inclusion criteria were applied to create a sample homogeneous with the Swiss cohort.) (PNG 324 kb) [file 192_2021_4864_MOESM5_ESM.png]

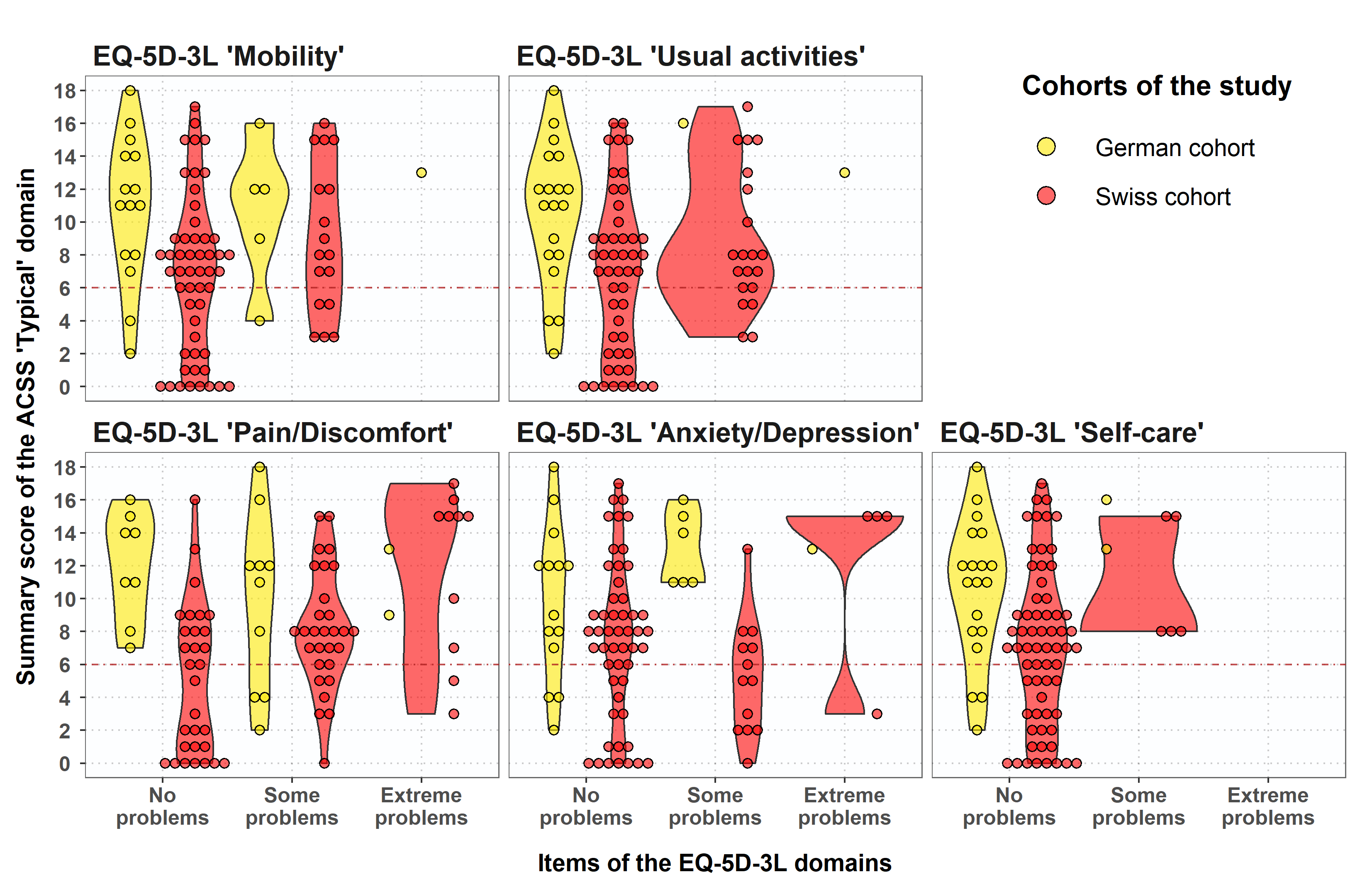

Supplement: Supplementary file 6 — (A-D) Relationships between the items of the validated German 3-level version of the EuroQoL-5 Dimension (EQ-5D-3L) Health Questionnaire and summary scores of: A: “Typical” domain, B: “Differential” domain, C: “QoL” domain of the ACSS and D: entire ACSS. (PNG 188 kb) [file 192_2021_4864_MOESM6_ESM.png]

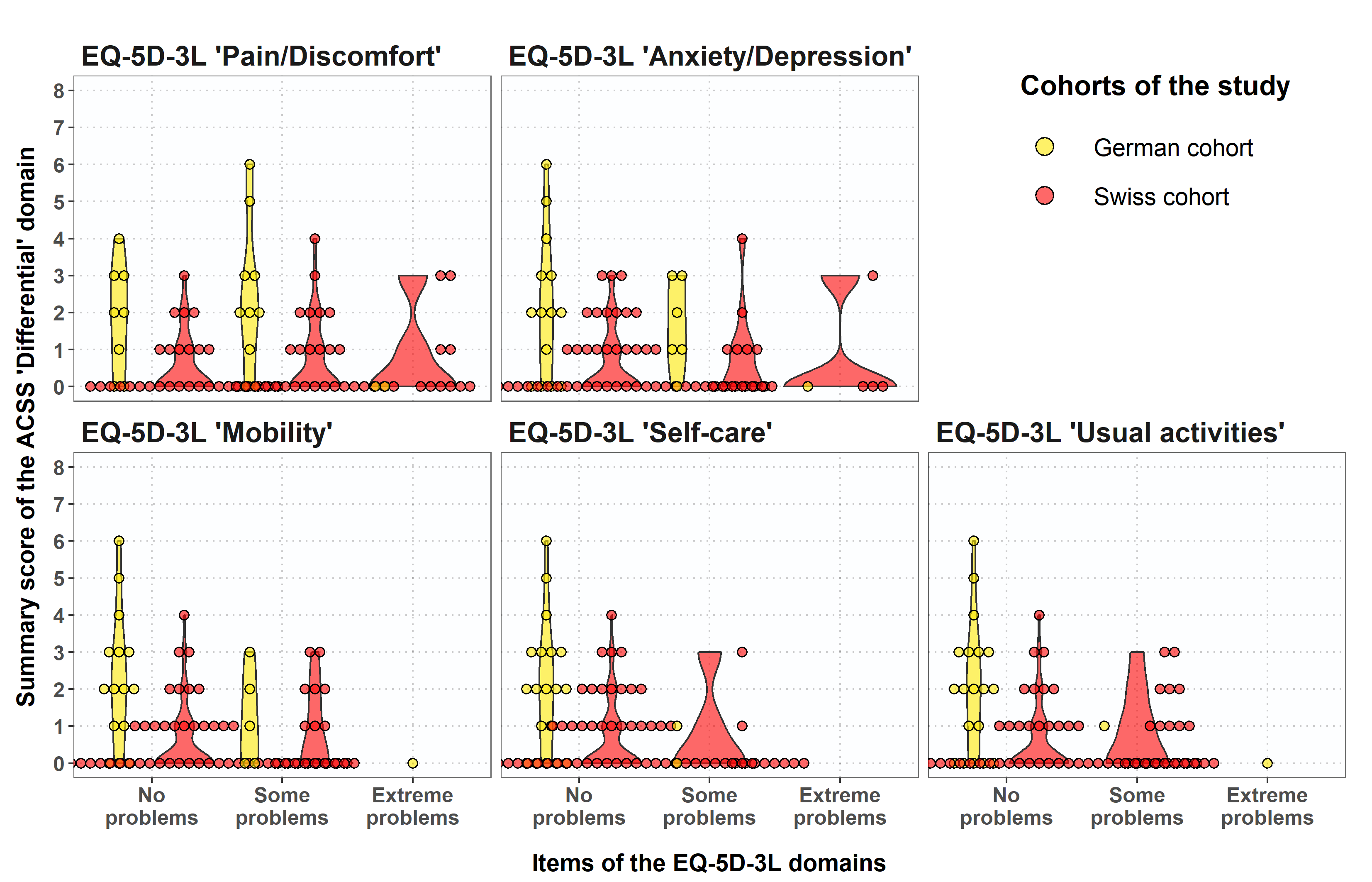

Supplement: Supplementary file 7 — (PNG 161 kb) [file 192_2021_4864_MOESM7_ESM.png]

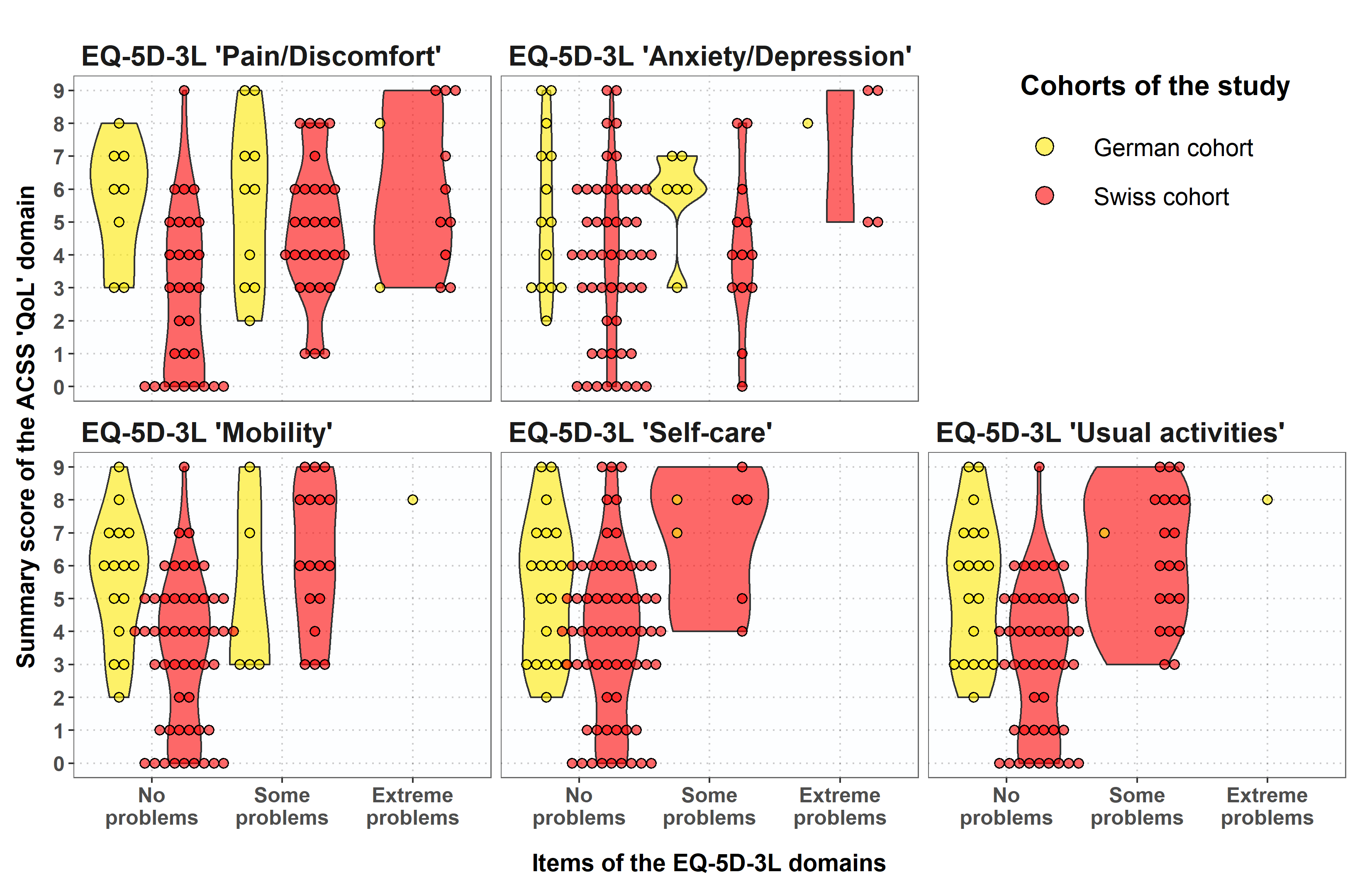

Supplement: Supplementary file 8 — (PNG 175 kb) [file 192_2021_4864_MOESM8_ESM.png]

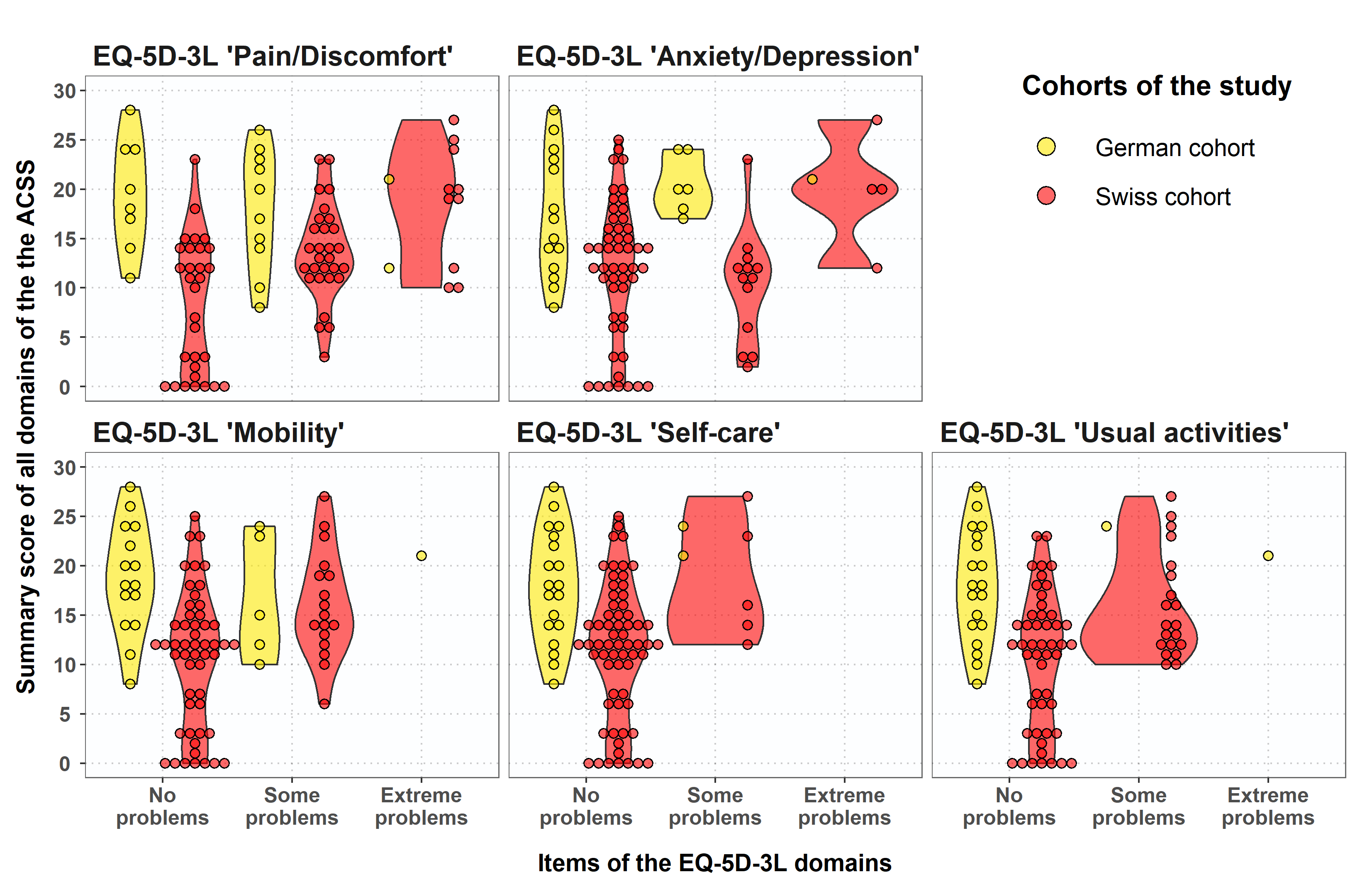

Supplement: Supplementary file 9 — (PNG 135 kb) [file 192_2021_4864_MOESM9_ESM.png]

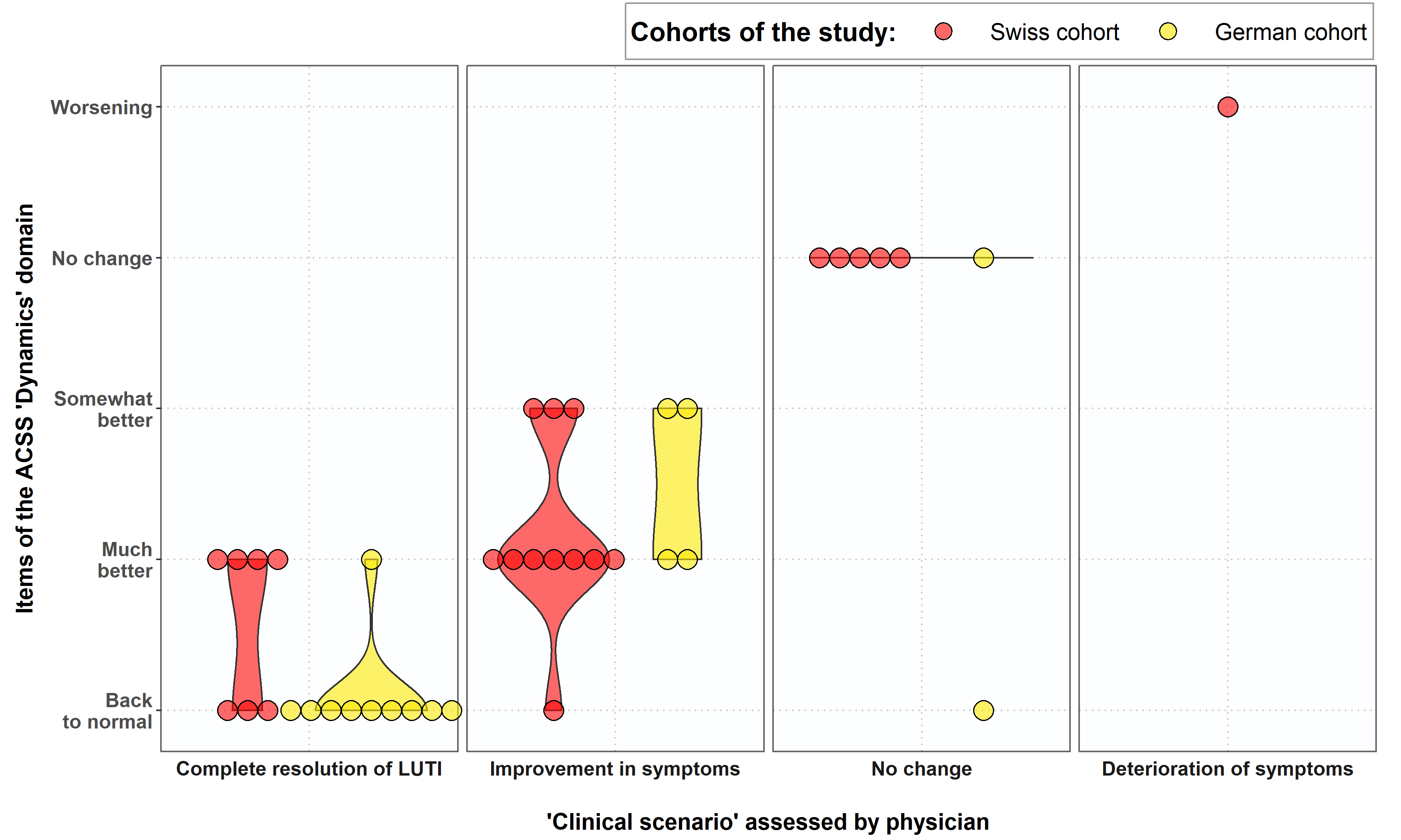

Supplement: Supplementary file 10 — Relationships between the items of the “Dynamics” domain of the ACSS and the “clinical scenario” assessed by the physician. (PNG 82 kb) [file 192_2021_4864_MOESM10_ESM.png]
